# IL-17A regulates autophagy and promotes osteoclast differentiation through the ERK/mTOR/Beclin1 pathway

**DOI:** 10.1371/journal.pone.0281845

**Published:** 2023-02-16

**Authors:** Hao Tang, Shida Zhu, Kai Chen, Shujie Yuan, Junzu Hu, Hongkai Wang

**Affiliations:** 1 Department of Orthopaedics, The Second Affiliated Hospital of Guilin Medical University, Guilin, Guangxi, China; 2 Department of Guangxi, Key Laboratory of Glucose and Lipid Metabolism, The Second Affiliated Hospital of Guilin Medical University, Guilin, Guangxi, China; Universite de Nantes, FRANCE

## Abstract

Bone is a frequent target of tumor metastasis, with high incidence rate and poor prognosis. Osteoclasts play a key role in the process of tumor bone metastasis. Interleukin-17A (IL-17A) is an inflammatory cytokine, highly expressed in a variety of tumor cells, that can alter the autophagic activity of other cells, thereby causing corresponding lesions. Previous studies have shown that low concentration IL-17A can promote osteoclastogenesis. The aim of this study was to clarify the mechanism of low concentration IL-17A promoting osteoclastogenesis by regulating autophagic activity. The results of our study showed that IL-17A could promote the differentiation of osteoclast precursors (OCPs) into osteoclasts in the presence of RANKL, and increase the mRNA levels of osteoclast-specific genes. Moreover, IL-17A increased the expression of Beclin1 by inhibiting the phosphorylation of ERK and mTOR, leading to enhanced autophagy of OCPs, accompanied by decreased OCP apoptosis. Furthermore, knockdown of Beclin1 and suppression of autophagy by 3-methyladenine (3-MA) significantly attenuated the enhanced osteoclastogenesis induced by IL-17A. In summary, these results indicate that low concentration IL-17A enhances the autophagic activity of OCPs through the ERK/mTOR/Beclin1 pathway during osteoclastogenesis, and further promotes osteoclast differentiation, suggesting that IL-17A may serve as a potential therapeutic target for cancer-related bone resorption in cancer patients.

## Introduction

Tumor bone metastasis can activate osteoclasts directly or indirectly, resulting in intractable pain, pathological fracture, nerve compression, anemia, hypercalcemia, and other complications [1], which seriously affect the quality of life in patients with cancer. Osteoclasts are bone-absorbing cells that secrete lysosomal enzymes into the extracellular space, resulting in the degradation of bone matrix. This can accelerate the occurrence of bone metastasis and related complications by destroying bone tissue. Tumor cells secrete various factors that stimulate osteoclast-mediated bone resorption, and there are studies showing that IL-1, IL-6, IL-8, tumor necrosis factor (TNF)-α, and transforming growth factor (TGF)-β are secreted by tumor cells and contribute to osteoclast-induced bone resorption [2–4]. However, effects of other factors derived from tumor cells on osteoclast differentiation are poorly understood.

IL-17 is a pro‑inflammatory cytokine typically secreted by T helper 17 (Th17) cells. The IL-17 cytokine family includes at least six members: IL-17A, IL-17B, IL-17C, IL-17D, IL-17E, and IL-17F; of which IL-17A is the main bioactive subtype. IL-17A is highly expressed in the serum of tumor patients and is closely related to the invasion and metastasis of tumor cells [3, 5, 6]. The effect of IL-17A on osteoclastogenesis depends on its concentration. A low concentration of IL-17A stimulates the differentiation of osteoclasts, while a high concentration of IL-17A shows the opposite effect [7, 8]. Nevertheless, the underlying mechanisms remain unclarified.

Autophagy, which is a highly conserved intracellular metabolic mechanism, can promote cell proliferation, differentiation, and prevent apoptosis [9]. Recently, autophagy was shown to play a role in osteoclast differentiation and bone resorption. Autophagy-related proteins, including Atg5, Atg7, Beclin1, and LC3 are responsible for forming ruffled borders and promoting osteoclast polarization which eventually lead to bone resorption [10]. Although it has been reported that in the process of RAW 264.7 induced osteoclastogenesis, low concentration IL-17A promotes osteoclastogenesis by increasing autophagy through the Beclin1 pathway [11, 12], it remains unclear how IL-17A affects osteoclast differentiation by regulating the autophagic activity of OCPs.

In the present study, RAW 264.7 cells and BMMs were used to study the mechanism of low concentration IL-17A affecting osteoclast differentiation by regulating autophagy. Our results indicate that IL-17A promotes osteoclastogenesis by activating autophagy of OCPs. Furthermore, we propose for the first time that ERK/mTOR/Beclin1 pathway may exert a pivotal role in low level IL-17A induced autophagy of OCPs.

## Materials and methods

### Cell culture

RAW 264.7 murine macrophages, obtained from the American Type Culture Collection (Manassas, VA, USA), were cultured at 37°C in a 5% CO_2_ atmosphere in Dulbecco’s modified Eagle medium (DMEM) supplemented with 10% fetal bovine serum (FBS) (Thermo Fisher Scientific, Waltham, MA, USA), 100 IU/ml penicillin, and 100 mg/ml streptomycin(Beyotime Biotechnology, Wuhan, Hubei, China). The culture medium was replaced every other day. RAW 264.7 cells were cultured in the presence of RANKL (50 ng/ml) in the process of osteoclastogenesis.

Bone marrow macrophages (BMMs) were obtained from 6-week-old male C57BL/6 mice. Mice were euthanized by cervical dislocation after being anesthetized with sodium pentobarbital (150 mg/kg body weight, intraperitoneally). The tibias and femurs of mice were dissected, and the marrow cavity was flushed with α-MEM medium containing 10% FBS. Cells were centrifuged for 5 min at 1000 rpm, and the cell pellet was resuspended in α-MEM supplemented with 10% FBS and cultured for 24 h. Non-adherent cells were collected and cultured for 3 days in the presence of macrophage colony-stimulating factor (M-CSF; 20 ng/ml) (PeproTech, Rocky Hill, NJ, USA). Floating cells were discarded, and adherent cells were classified as BMMs. BMMs were cultured in the presence of RANKL (50 ng/ml) and M-CSF (20 ng/ml) in the process of osteoclastogenesis.

All animal experiments were performed in accordance with the principles and procedures of the National Institutes of Health (NIH) for the Care and Use of Laboratory Animals. The approval number granted by the Animal Ethics Committee of Guilin Medical University is GLMC-IACUC-2022017. All surgeries were performed under sodium pentobarbital anesthesia, and all efforts were made to minimize suffering.

### Cytotoxicity assay

Cell viability was examined using a Cell Counting Kit (CCK)-8 (Dojindo Laboratories, Kumamoto, Japan). The cells (2×10^3^ cells/well) were seeded into 96-well plates and cultured with 3-Methyladenine (3-MA) (Aladdin, Shanghai, China) of different concentrations for 48h. 3-MA is an autophagic inhibitor widely used in autophagy related research. After incubation, 10 μl CCK-8 solution was added. The plates were incubated at room temperature for 4 h before measuring the absorbance at 450 nm using a microplate reader (Bio-Rad, Hercules, CA, USA).

### RNA interference

An siRNA designed to target mouse Beclin1 was synthesized by OBiO Technology (Shanghai, China). The target sequences were as follows: 5’-GCTGGACACTCAGCTCAAT-3’ (control) and 5’-GCTACACTCCGACTGGAAT-3’ (Beclin1). siRNAs were transfected into OCPs using Lipofectamine 3000 reagent (Thermo Fisher Scientific), according to the manufacturer’s instructions [13]. After 6 h, the medium was replaced with fresh DMEM supplemented with 10% heat-inactivated FBS. The siRNA transfection efficiency was detected by western blotting 24 h after transfection.

### Tartrate-resistant acid phosphatase (TRAP) staining of RAW 264.7 cells and BMMs

To confirm the generation of multinucleated osteoclast like cells, the cultured cells were stained for tartrate-resistant acid phosphatase (TRAP) using the TRAP Staining Kit (Sigma-Aldrich, St Louis, MO, USA), as previously described [13]. TRAP-positive multinucleated cells with three or more nuclei were considered as osteoclasts, and the number of osteoclasts in each well was counted using ImageJ software. All TRAP staining experiments were completed with 24-well plates, and at least four repeat wells were set for each experiment.

### RNA isolation and real-time PCR analysis

Total RNA was isolated from the cells using TRIzol Reagent (Thermo Fisher Scientific), according to the manufacturer’s instructions. Equal amounts of total RNA from each sample were reverse-transcribed into cDNA using the PrimeScript RT Reagent Kit with cDNA Eraser (Takara Bio, Kusatsu, Shiga, Japan). Real-time PCR was conducted using FastStart Universal SYBR Green Master Mix (Roche GmbH, Mannheim, Germany) and the expression levels were normalized to that of GAPDH. Primer sequences can be found in S1 Table.

### Western blot analysis

RAW 264.7 cells or BMMs were cultured for 24 h in the presence of RANKL (PeproTech, Rocky Hill, NJ, USA), with or without IL-17A (R&D Systems, Minneapolis, MN, USA), and then whole-cell proteins were extracted using radioimmunoprecipitation assay buffer (Beyotime Biotechnology, Wuhan, Hubei, China) supplemented with a cocktail of protease inhibitors (Roche, Basel, Switzerland). Cell lysates were centrifuged at 12,000 rpm for 30 min, after which supernatants were collected. Proteins were separated by 8% SDS-PAGE and transferred to polyvinylidene difluoride membranes. After transfer, the membranes were blocked with 5% BSA for 2 h in PBS with 0.1% Triton X-100 before incubating with the primary antibodies overnight at 4°C. After rinsing with PBST buffer, the membranes were incubated with secondary antibodies for 2 h at room temperature. The immunoreactive bands were visualized using an ECL kit (Epizyme Biomedical Technology, Shanghai, China), and the data were analyzed using ImageJ software. The used primary antibodies against LC3, Beclin1, ATG5, ATG7, p-mTOR, p-Erk, cleaved caspase-3 and β-Actin were obtained from Cell Signaling Technology (Danvers, MA, USA), and the secondary antibodies were obtained from Boster Bio (Wuhan, Hubei, China). At minimum, the experiments were performed in triplicate. Expression of proteins was normalized against β-Actin.

### Electron microscopy

Cells were provided with the indicated treatments, centrifuged to the bottom of the tube after trypsin digestion, subsequently washed once in PBS, and then fixed with 2.5% glutaraldehyde at 4°C for 1 h. After five times of washing with PBS, cells were post-fixed in 1% buffered osmium tetroxide at 20°C for 2 h. Then samples were dehydrated to 100% through ethanol and embedded in epoxy resin using Epoxy embedding medium kit (Sigma-Aldrich, St Louis, MO, USA) overnight, baked in a 60°C oven for 48 h and cut into ultrathin sections (70 nm). Subsequently, the sections were stained with 2% uranyl acetate for 20 min and lead citrate for 10 min, and observed using Hitachi 7700 Transmission EM (Tokyo, Japan). At least 60 cells were observed, and the number of autolysosomes was counted using ImageJ software. The experiments were replicated for at least three times.

### Cell apoptosis analysis

After treatment with or without IL-17A, cells were collected and suspended in PBS. Cells were taken into tubes and suspended in binding buffer, then stained using an Annexin V‑FITC/PI apoptosis detection kit (Beyotime Biotechnology, Wuhan, Hubei, China) and evaluated using a flow cytometer (BD Bioscience, Sparks, Maryland, USA). The experiments were replicated for at least three times.

### Statistical analysis

At minimum, all experiments were performed in triplicate. Student’s t-test was used to determine the significance of differences between the two groups, while one-way analysis of variance and Tukey’s test were used for multiple comparisons using SPSS Statistics 20.0 (IBM Corp., Armonk, NY, USA). All data were presented as mean ± SD. P values less than 0.05 (p<0.05) were considered statistically significant.

## Results

### IL-17A promotes RANKL-stimulated osteoclastogenesis

**Fig 1:**
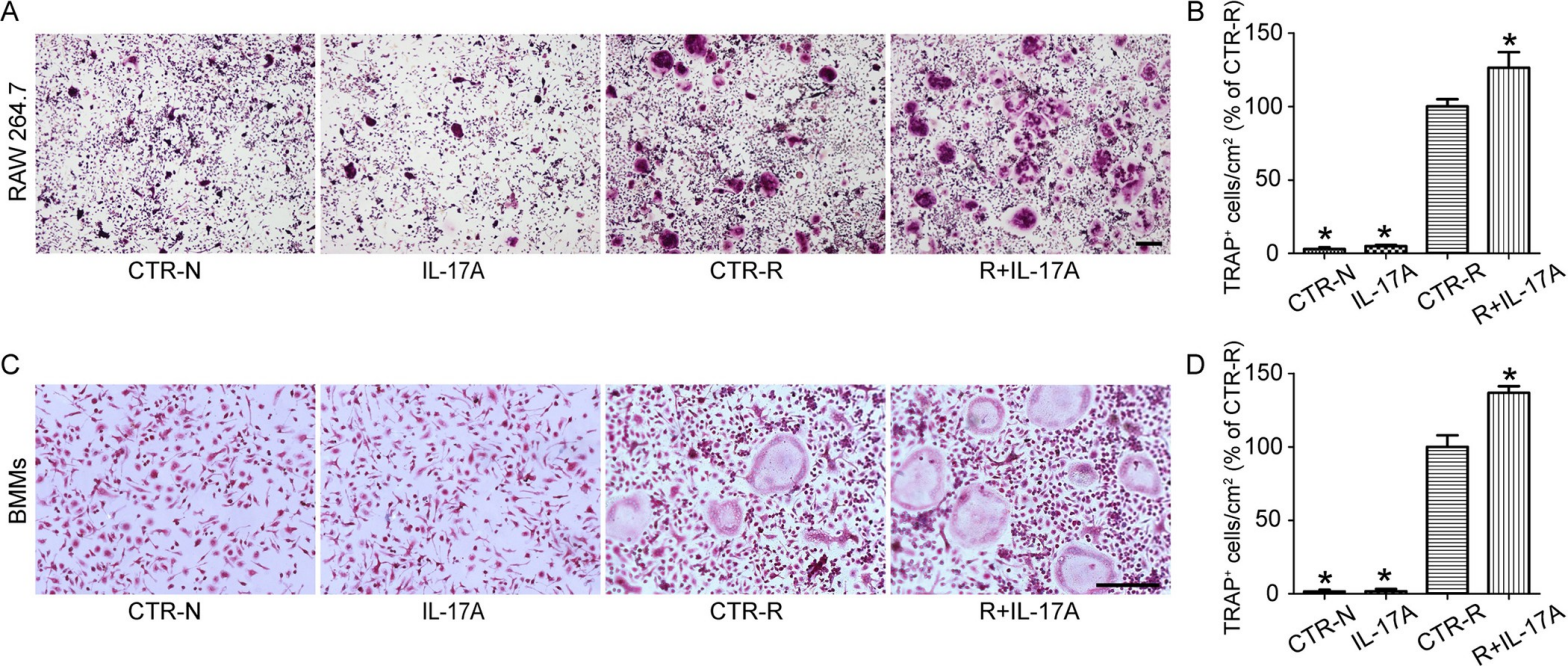
IL-17A promotes RANKL-stimulated osteoclastogenesis. (A) RAW 264.7 cells and (C) BMMs were cultured with or without IL-17A (1 ng/ml) in the presence of RANKL (50 ng/ml) for 5 d. (B, D) TRAP-positive multinucleated cells with three or more nuclei were considered as osteoclasts, and the number of osteoclasts were counted in each well. At least four wells were used for each tested reagent. Cells treated without RANKL and IL-17A (CTR-N) were used as a negative control. Cells not treated with IL-17A but exposed to RANKL (CTR-R) were used as a positive control. The values are expressed as the mean ± SD. *p < 0.05 compared with RANKL treatment. Scale bar, 200 μm.

To detect the effect of IL-17A on osteoclastogenesis, RAW 264.7 cells were cultured in the presence of RANKL (50 ng/ml) for 5 d, with and without IL-17A (1 ng/ml). The formation of TRAP-positive multinucleated cells marked the differentiation of osteoclasts. The staining results revealed an increase in TRAP-positive multinucleated osteoclasts among RAW 264.7 cells exposed to RANKL (50 ng/ml); the increase in the number of TRAP-positive multinucleated osteoclasts was more significant when IL-17A was added. However, IL-17A did not induce osteoclast differentiation in the absence of RANKL (Fig 1A and 1B). To further evaluate the effect of IL-17A on osteoclastogenesis, BMMs were treated with or without IL-17A (1 ng/ml) in the presence of RANKL (50 ng/ml) and M-CSF (20 ng/ml) for 5 d. TRAP staining showed that IL-17A also promoted the differentiation of osteoclasts derived from BMMs (Fig 1C and 1D).

### IL-17A promotes the expression of osteoclast-related genes

**Fig 2:**
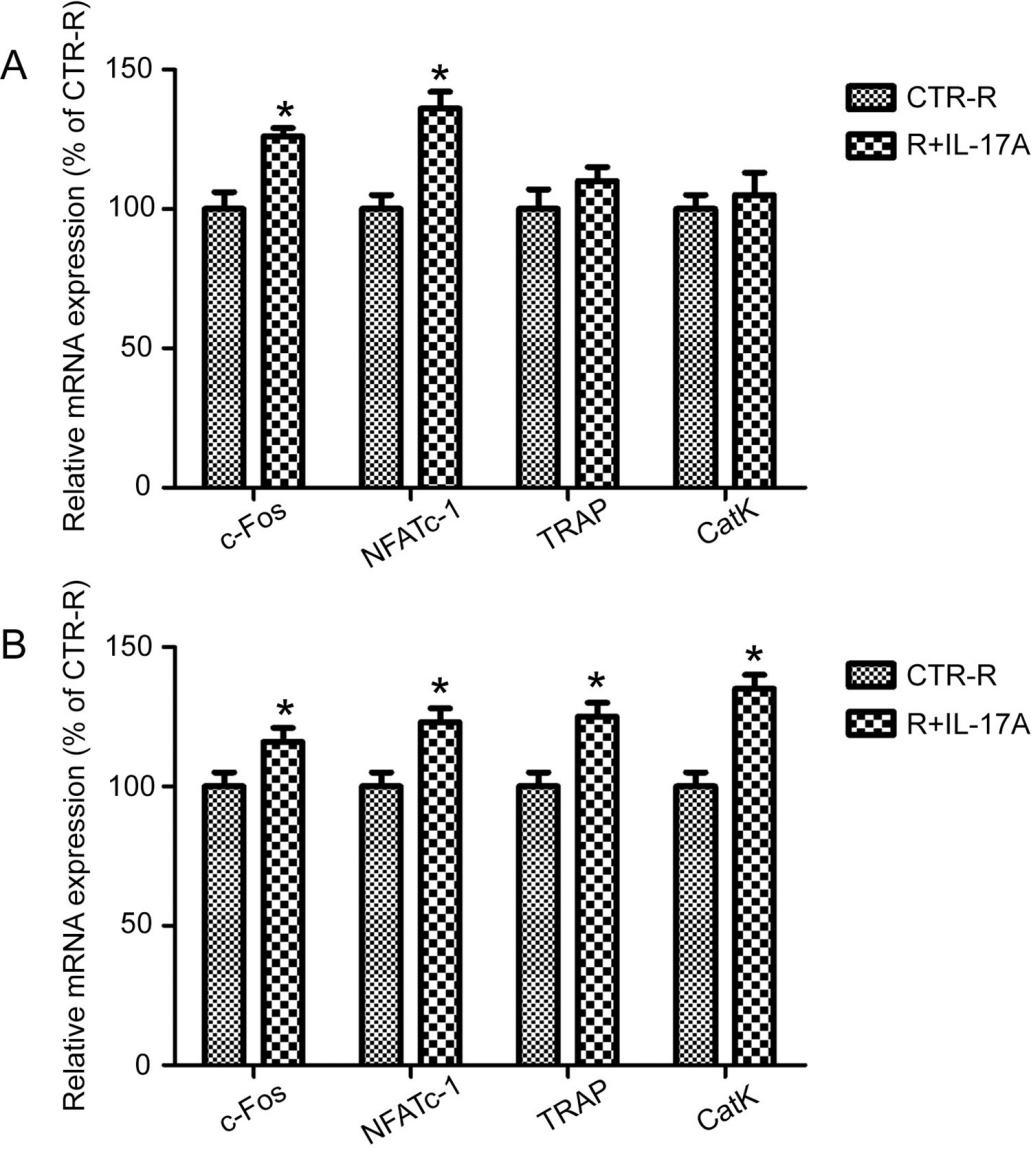
IL-17A promotes the expression of osteoclast-related genes involved in osteoclastogenesis. The mRNA expression levels of *c-Fos*, *NFATc1*, *TRAP*, and *CatK* were detected in the presence of RANKL (50 ng/ml) after IL-17A (1 ng/ml) treatment for (A) 1 and (B) 3 d. Data represent fold-changes in target gene expression normalized to that of GAPDH and are expressed as a percentage of the level in cells not treated with IL-17A (CTR-R), which was set to 100%. Values represent the mean ± SD (n = 4). *p < 0.05.

To further study the effects of IL-17A on osteoclast differentiation, real-time quantitative PCR was used to detect the expression of osteoclast-related genes at different time points. In the process of differentiation, osteoclasts express a group of markers, including *c-Fos*, *NFATc1*, *TRAP*, and *CatK*; these markers characterize the osteoclast phenotype along with multinucleation and resorption [14, 15]. The expression of osteoclast-related genes in RAW264.7 cells treated with and without IL-17A (1ng/ml) was examined after 1 and 3 d. The expression of *c-Fos* and *NFATc1* increased slightly in the early stage of osteoclast differentiation (Fig 2A). Compared with the control group, the IL-17A treatment group showed a considerable increase in the expression of *TRAP* and *CatK* at the late stage of osteoclast differentiation (Fig 2B).

### ERK/mTOR/Beclin1 signal mediates IL-17A-promoted OCP autophagy

**Fig 3:**
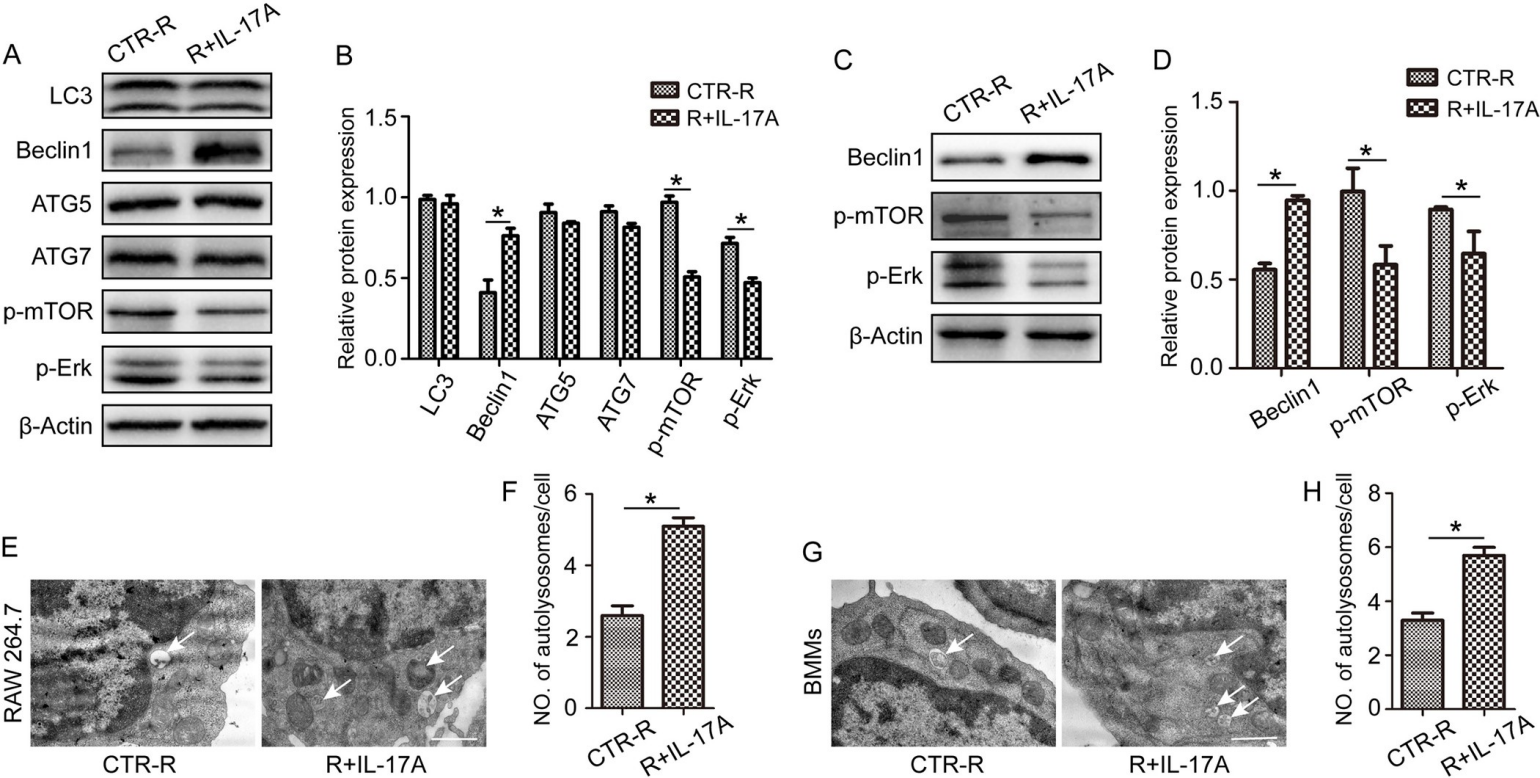
IL-17A promotes OCP autophagy through the ERK/mTOR/Beclin1 pathway induced by RANKL. RAW 264.7 cells and BMMs were stimulated with or without IL-17A (1 ng/ml) in the presence of RANKL (50 ng/ml) for 24 h. (A, C) Autophagy-related proteins were detected by western blotting and (B, D) protein expression was normalized against β-Actin. (E, G) TEM images and (F, H) numbers of autolysosomes (arrows) in RAW 264.7 cells and BMMs after treatment with or without IL-17A (1 ng/ml) for (A) 1 d in the presence of RANKL (50ng/ml). At least 60 cells were observed. Cells not treated with IL-17A but exposed to RANKL (CTR-R) were used as control. Data are presented as the mean ± SD from three independent experiments. *p < 0.05. Scale bar, 1 μm.

Autophagy plays an important part in osteoclast differentiation and the specific mechanism by which IL-17A promotes osteoclast differentiation is hypothesized to be the autophagic pathway. Therefore, the expression of autophagy markers LC3, Beclin1, Atg5, and Atg7 was investigated in RAW264.7 cells after IL-17A treatment for 24 h. Western blot analysis showed that the expression of Beclin1 increased after the addition of IL-17A. Furthermore, after IL-17A treatment, the expression of p-ERK and p-mTOR decreased, but the changes in LC3, Atg5, and Atg7 levels were not significant (Fig 3A and 3B). In addition, we further verified the effect of IL-17A on the expression of Beclin1, p-ERK and p-mTOR in BMMs. Western blot results showed that IL-17A also promoted the expression of Beclin1 and inhibited the expression of p-ERK and p-mTOR (Fig 3C and 3D). Moreover, the electron microscopic results showed that IL-17A could increase the number of autolysosome in both RAW264.7 cells and BMMs (Fig 3E and 3F).

### IL-17A inhibits apoptosis activity during osteoclast differentiation

**Fig 4:**
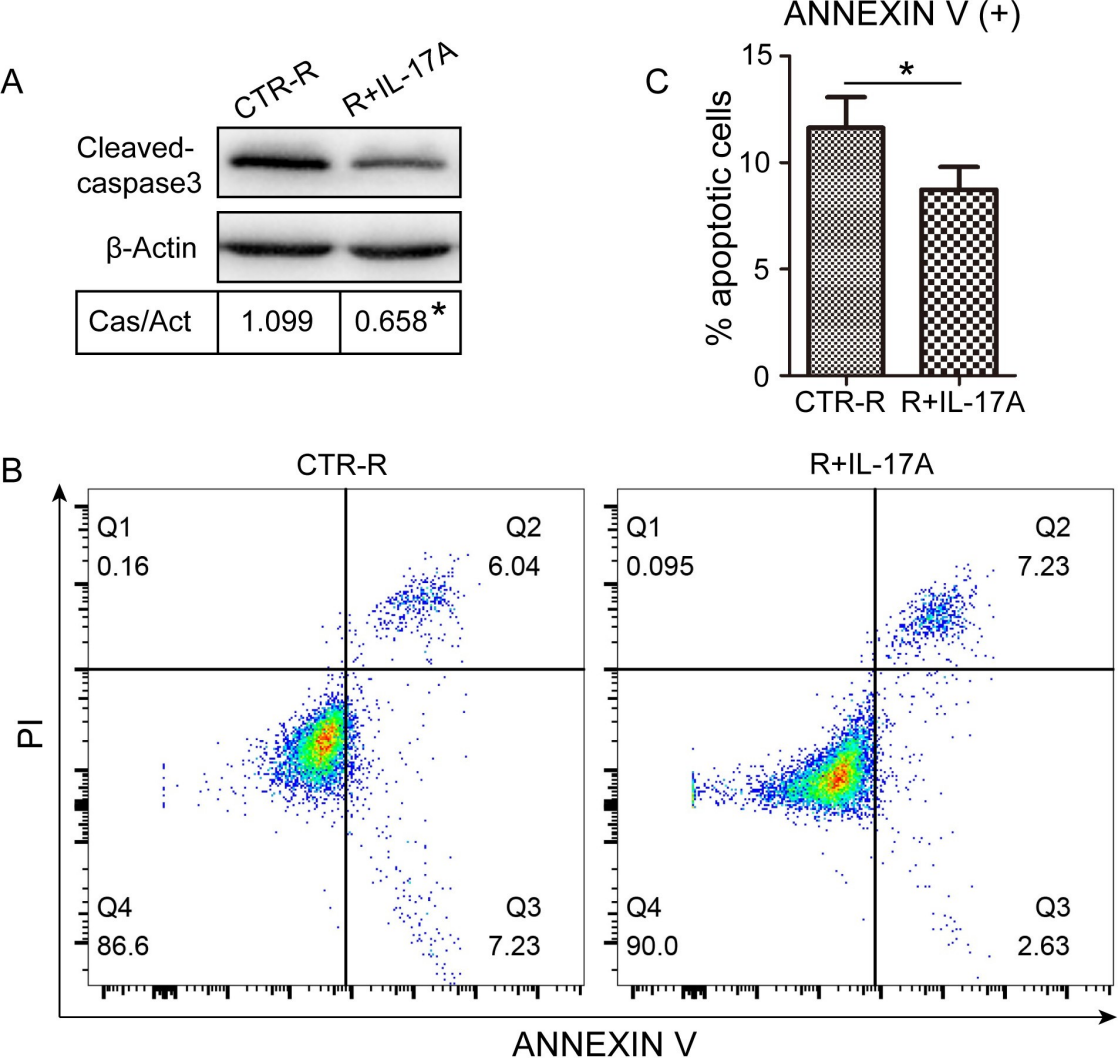
IL-17A inhibits apoptosis of OCPs. RAW264.7 cells were treated with or without IL-17A (1 ng/ml) in the presence of RANKL (50 ng/ml) for 24 h, cleaved caspase-3 was detected by western blotting (A), and Annexin V-FITC/PI staining was performed to label apoptotic cells. The percentages of apoptotic cells (Annexin V+) were counted by flow cytometry (B), Q2 and Q3 quadrants indicated reduced apoptosis with IL-17A treatment (C). Protein expression was normalized against β-Actin. Cells not treated with IL-17A but exposed to RANKL (CTR-R) were used as control. Data are presented as the mean ± SD from three independent experiments. *p < 0.05.

To clarify the effect of IL-17A on apoptosis of OCPs, RAW264.7cells were treated with or without IL-17A (1 ng/ml) for 24 h in the presence of RANKL (50ng/ml). Western blot analysis showed that IL-17A inhibited the expression of apoptotic protein cleaved-caspase3 (Fig 4A). In addition, flow cytometry was used to further verify the effect of IL-17A on OCP apoptosis. The results showed that the number of Annexin V positive cells in RAW264.7 cells treated with IL-17A decreased significantly (Fig 4B and 4C). These findings indicate that IL-17A can inhibit the apoptosis of OCPs, which may be related to the inhibition of IL-17A on the expression of cleaved-caspase3.

### Inhibition of autophagy attenuates IL-17A-mediated osteoclastogenesis

**Fig 5:**
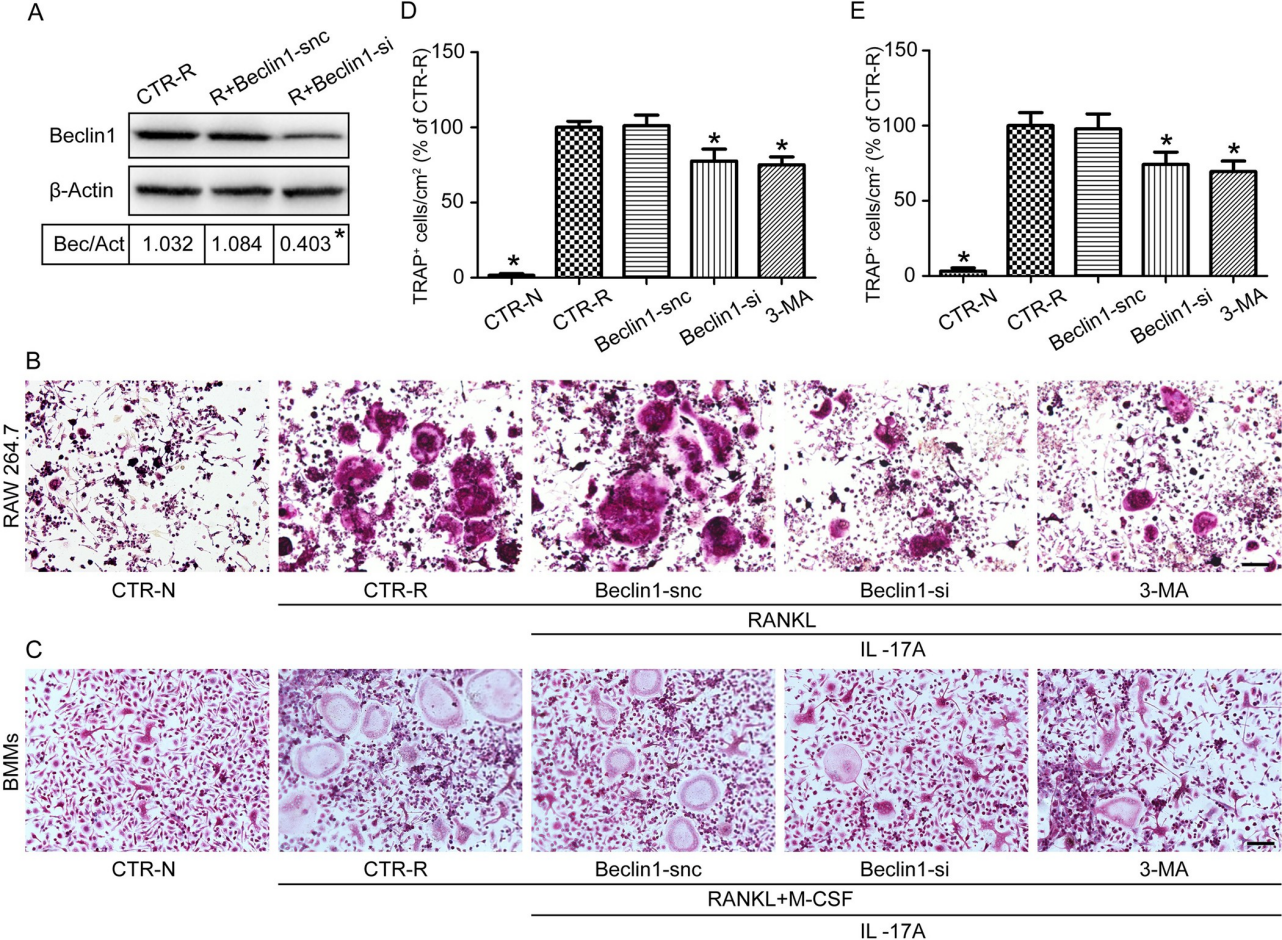
Inhibition of autophagy attenuates IL-17A-mediated osteoclastogenesis. A specific siRNA targeting Beclin1 mRNA was used to knockdown Beclin1 expression. (A) At 24 h post-transfection, the siRNA transfection efficiency was detected by western blotting. Protein expression was normalized against β-Actin. RAW264.7 cells and BMMs were treated with Beclin1-si or 3-MA in the presence of IL-17A (1 ng/ml) and RANKL (50 ng/ml) for 5 d (B, C) and following treatment, TRAP-positive multinucleated cells were quantified (D, E). At least four wells were used for each tested reagent. Cells treated without RANKL and IL-17A (CTR-N) were used as a negative control. Cells treated with RANKL and IL-17A (CTR-R) were used as a positive control and the values are expressed as the mean ± SD. *p < 0.05 compared with CTR-R. Scale bar, 100 μm.

To further verify the positive role of Beclin1 in IL-17A-treated OCPs, a specific siRNA targeting Beclin1 mRNA was used to knock down the expression of Beclin1. An autophagy inhibitor, 3-MA (5mM), was used to inhibit autophagy during osteoclast differentiation and the toxic effect of 3-MA on OCPs was detected by CCK-8 (S1 Fig). Protein expression of Beclin1 showed significant downregulation as a result of Beclin1-siRNA-mediated knockdown, as detected by western blotting (Fig 5A). After five days of treatment with Beclin1-si, or MA, in the presence of IL-17A (1 ng/ml) and RANKL (50 ng/ml), the number of TRAP+ multinuclear cells decreased in IL-17A-treated RAW264.7 cells compared to controls (Fig 5B and 5D). The same results were observed in BMMs (Fig 5C and 5E).

## Discussion

Nearly 90% of tumor-related deaths can be attributed to tumor metastasis; mainly to lungs, lymph nodes, liver, and bone. The latter is often seen in lung, prostate, and breast cancer. Tumor metastasis to the bone always results in osteolytic lesions and osteoclast differentiation is a critical event in bone loss induced by tumor [16, 17]. Tumor cells can secrete various factors that promote the differentiation of osteoclasts and aid the progression of bone metastasis [2, 18]. Pro-inflammatory cytokine IL-17A is highly expressed in a variety of tumor cells [5, 6]. Previous studies have shown that osteoclastogenesis is promoted at low concentrations and inhibited at high concentrations of IL-17A [8]. Xue et al. [11] demonstrated that different concentrations of IL-17A affect osteoclastogenesis by regulating autophagy and apoptosis of OCPs through autophagy-TRAF3 signal pathway in RAW264.7 induced osteoclastogenesis. Nevertheless, another study showed that with the increase of IL-17A concentration, the promotion of IL-17A on osteoclast differentiation was gradually enhanced in BMMs induced osteoclastogenesis [19]. Therefore, theories on the activity of IL-17A in osteoclast differentiation currently remain controversial and it is imperative to explore the specific potential mechanisms.

Considering the correlation between IL-17A and the poor prognosis of tumor patients, and the promotion of low concentration IL-17A on osteoclastogenesis shown in previous studies, in this study, RAW264.7 cells and BMMs were used to focus on the effect of low concentration IL-17A on osteoclastogenesis and the mechanism involved. Our results revealed that IL-17A promoted the differentiation of osteoclasts in the presence of RANKL, while IL-17A alone could not promote osteoclast differentiation, indicating that the promoting effect of IL-17A on osteoclastogenesis is attributed to the synergistic effect of IL-17A and RANKL, which is consistent with the results reported by Adamopoulos et al. [8] and Ke et al. [12].

Osteoclast-specific genes participate in various stages of osteoclast formation and activation [15]. *c-Fos* is necessary at the early phase of osteoclast differentiation [20] and *NFATc1* is critical for survival and differentiation of osteoclast precursors [21]. *TRAP* and *CatK* are highly expressed by activated osteoclasts and mediate bone resorption [22, 23]. In this study, the expression of *c-Fos*, *NFATc1*, *TRAP*, and *CatK* was detected after 1 and 3 days of treatment in RAW264.7 cells. The results showed that, in comparison with the control group, IL-17A promoted the expression of *c-Fos* and *NFATc1* during both the early and the late stages of osteoclast differentiation, whereas *TRAP* and *CatK* were highly expressed during the late stage, indicating that IL‑17A could promote osteoclast differentiation in RAW264.7 cells. The result above is consistent with the study by Song et al. [19]. They found that the expression of c‑Fos, NFATc1, CatK, and TRAP genes was increased with the treatment of IL‑17A. However, Kitami et al. [7] observed that both low and high concentrations of IL-17A inhibited osteoclast differentiation and CatK gene expression, which was inconsistent with most of the current research results. Different experimental results may be attributed to various factors, the most common of which is different experimental conditions.

As a protective mechanism, autophagy plays a crucial role in cell survival and can promote osteoclast proliferation and differentiation, as well as bone resorption [10, 24]. New evidence suggests that autophagy is activated in response to several pro-inflammatory cytokines and is accompanied by a significant increase in osteoclast differentiation and bone resorption [25]. In addition, the effects of IL-17A on autophagy have been elucidated in several studies, although they were found to be inconsistent [26]. Accumulating evidence has revealed that Beclin1, a key protein in response to autophagy activity, plays an important role in osteoclastogenesis [27]. Zhao et al. [28] revealed that NUCKS promoted cell proliferation and suppressed autophagy through the mTOR-Beclin1 pathway in gastric cancer. Lu et al. [29] also reported the negative correlation between Beclin-1 and mTOR protein expression in malignant epithelial ovarian tumor. Our results indicated that IL-17A promoted Beclin1 expression and inhibited ERK and mTOR phosphorylation, accompanied by an increase in the number of autolysosome in both RAW264.7 cells and BMMs. Further studies demonstrated that suppression of autophagy with 3-MA or Beclin1 silencing inhibited the effect of IL-17A on osteoclast differentiation; further verifying that IL-17A affects osteoclast differentiation by regulating autophagy. For the first time, we proposed that IL-17A promoted OCP autophagy through the ERK/mTOR/Beclin1 pathway in osteoclastogenesis. Similarly, Jung et al. [30] reported that Chrysin induced autophagy through the ERK/mTOR pathway in MC-3 oral cancer cells.

As a highly conserved intracellular metabolic mechanism, autophagy is widely accepted to have anti-apoptotic effects [31]. Studies have shown that caspase-3 activation inhibits RANKL-induced osteoclast differentiation in the late stage [32]. In this study, IL-17A inhibited the activation of caspase-3. OCPs treated with IL-17A also showed a significant decrease in the number of apoptotic cells, as determined using Annexin V-FITC/PI staining. A similar viewpoint has also been elucidated by Xue et al. [11]. They observed that IL-17A regulated the expression of caspase-3 through Beclin1-autophagy-TRAF3 signaling pathway and further affected the apoptosis of OCPs, thereby influencing osteoclastogenesis.

In summary, RAW264.7 cells were often used as OCPs to observe the effect of IL-17A on osteoclast differentiation in previous studies [8, 11, 12]. In this study, RAW264.7 cells and BMMs were simultaneously used to more comprehensively observe the close relationship between autophagic activity in the presence of RANKL and IL-17A-mediated osteoclast differentiation. Our results indicate that IL-17A promotes osteoclastogenesis by activating autophagy in both RAW264.7 cells and BMMs. Furthermore, this study demonstrates for the first time that ERK/mTOR/Beclin1 pathway may exert a pivotal role in low level IL-17A induced autophagy of OCPs, which offers insights into the clinical therapeutic targets of bone metastasis. Limitation of this study is the use of IL-17A concentration and the selection of experimental observation time point, which are relatively simple. Therefore, these preliminary studies are insufficient to fully clarify the complex relationship between IL-17A and osteoclast differentiation. In addition, this study lacks in vivo research to further verify the experimental results. Considerations for the future work consist in carrying out more researches on osteoclast differentiation at different time points with different concentrations of IL-17A, and observing the effect of IL-17A on the survival of mature osteoclasts and bone resorption function. Meanwhile, it is our further work to verify the effect of IL-17A on osteoclast differentiation in vivo by using tumor bone metastasis animal model.

## Supporting information

S1 Fig Effect of 3-MA on cell viability. (PDF)

S1 Table Sequences of primers used in RT-PCR analysis. (PDF)
