# Supplementary material for: IL-17A regulates autophagy and promotes osteoclast differentiation through the ERK/mTOR/Beclin1 pathway
Source: PLoS One. 2023 Feb 16;18(2):e0281845. doi: 10.1371/journal.pone.0281845 (PMC9934321; doi:10.1371/journal.pone.0281845)
Supplement: S1 Fig — (PDF) [file pone.0281845.s001.pdf]

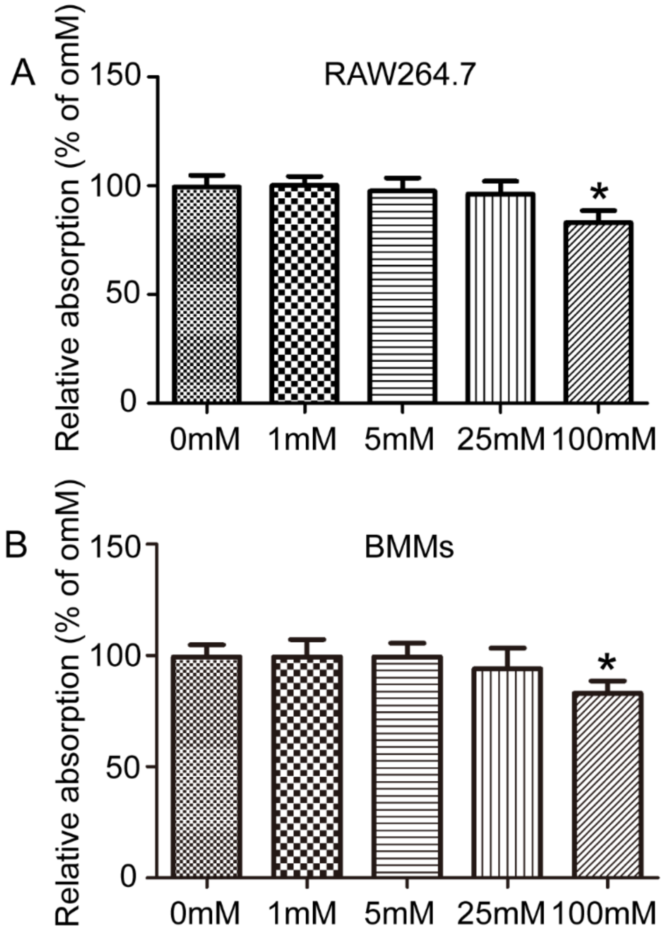

S1 Figure. Effect of 3-MA on cell viability. CCK-8 assay was performed after incubation of RAW 264.7 cells (A) and BMMs (B) with 3-MA (0, 1, 5, 25, and 100 mM) for 48 h. Data are presented as the mean ± SD of triplicate experiments. *$p < 0.05$.
