# Supplementary material for: IL-17A regulates autophagy and promotes osteoclast differentiation through the ERK/mTOR/Beclin1 pathway
Source: PLoS One. 2023 Feb 16;18(2):e0281845. doi: 10.1371/journal.pone.0281845 (PMC9934321; doi:10.1371/journal.pone.0281845)
Supplement: S1 Table — (PDF) [file pone.0281845.s002.pdf]

S1 Table. Sequences of primers used in RT-PCR analysis

Gene	Primer sequence (5'-3')
c-Fos	F: CGG GTT TCA ACG CCG ACT A
	R: TTG GCA CTA GAG ACG GAC AGA
NFATc1	F: GGA GAG TCC GAG AAT CGA GAT
	R: TTG CAG CTA GGA AGT ACG TCT
TRAP	F: GCA GTA TCT TCA GGC GAG AAC
	R: TCC ATA GTG AAA CCG CAA GTA G
Catk	F: TAT GAC CAC TGC CTT CCA ATA C
	R: GCC GTG GCG TTA TAC ATA CA
GAPDH	F: GGA GAA ACC TGC CAA GTA TGA
	R: TCC TCA GTG TAG CCC AAG A
